# *Bacillus subtilis* IolQ (DegA) is a transcriptional repressor of *iolX* encoding NAD^+^-dependent *scyllo*-inositol dehydrogenase

**DOI:** 10.1186/s12866-017-1065-8

**Published:** 2017-07-11

**Authors:** Dong-Min Kang, Christophe Michon, Tetsuro Morinaga, Kosei Tanaka, Shinji Takenaka, Shu Ishikawa, Ken-ichi Yoshida

**Affiliations:** 1 Department of Agrobioscience, Graduate School of Agricultural Science, Kobe University, 1-1 Rokkodai, Nada, Kobe, 657-8501 Japan; 2 Gene testing Business Department, LS Business Division, Sysmex Corporation, 4-4-4 Takatsukadai, Nishi, Kobe, 651-2271 Japan; 3 Organization of Advanced Science and Technology, Kobe University, 1-1 Rokkodai, Nada, Kobe657, Kobe, -8501 Japan; 4 Department of Science, Technology and Innovation, Graduate School of Science, Technology and Innovation, Kobe University, 1-1 Rokkodai, Nada, Kobe, 657-8501 Japan; 5 Present address: Department of Plant Medicine and RILS, Gyeongsang National University, Jinju, 52828 Republic of Korea

**Keywords:** *Bacillus subtilis*, *scyllo*-inositol, Inositol dehydrogenase, Transcription, Repressor

## Abstract

**Background:**

*Bacillus subtilis* is able to utilize at least three inositol stereoisomers as carbon sources, *myo*-, *scyllo*-, and D-*chiro*-inositol (MI, SI, and DCI, respectively). NAD^+^-dependent SI dehydrogenase responsible for SI catabolism is encoded by *iolX*. Even in the absence of functional *iolX*, the presence of SI or MI in the growth medium was found to induce the transcription of *iolX* through an unknown mechanism.

**Results:**

Immediately upstream of *iolX*, there is an operon that encodes two genes, *yisR* and *iolQ* (formerly known as *degA*), each of which could encode a transcriptional regulator. Here we performed an inactivation analysis of *yisR* and *iolQ* and found that *iolQ* encodes a repressor of the *iolX* transcription. The coding sequence of *iolQ* was expressed in *Escherichia coli* and the gene product was purified as a His-tagged fusion protein, which bound to two sites within the *iolX* promoter region in vitro.

**Conclusions:**

IolQ is a transcriptional repressor of *iolX.* Genetic evidences allowed us to speculate that SI and MI might possibly be the intracellular inducers, however they failed to antagonize DNA binding of IolQ in in vitro experiments.

## Background

**Fig. 1:**
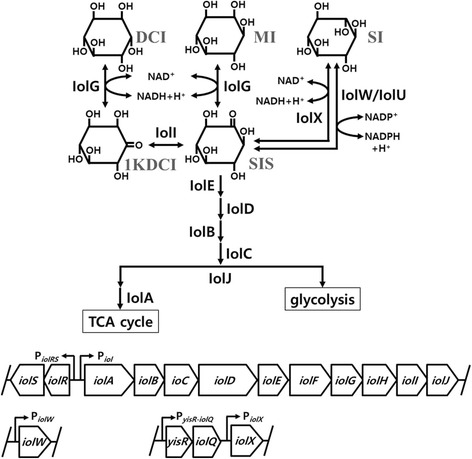
Inositol metabolic pathway in *Bacillus subtilis* (top) and organization of the relevant genes (bottom). D-*chiro*- (DCI), *myo*- (MI), and *scyllo*-inositols (SI) were converted to *scyllo*-inosose (SIS) and degraded further via the metabolic pathway involving the series of Iol enzymes. 1KDCI, 1-keto-D-*chiro*-inositol

Epimerization of the hydroxyl groups of cyclohexane 1,2,3,4,5,6-hexol (inositol) generates nine different stereoisomers. The most abundant form in nature is cis-1,2,3,5-trans-4,6-cyclohexanehexol (*myo*-inositol, MI) (Fig. 1), which is an essential component of phosphatidylinositol in the cell membranes of eukaryotes and exists as *myo*-inositol hexakisphosphate (phytic acid) in plant seeds [1]. Other inositol stereoisomers occur rarely in nature, although some exert specific and physiologically important effects. For example, D-*chiro*-inositol (DCI) (Fig. 1) and its 3-*O*-methyl derivative, D-pinitol, are beneficial for patients with hyperglycemia or polycystic ovary syndrome [2, 3], and *scyllo*-inositol (SI) (Fig. 1) directly interacts with beta-amyloid peptides to inhibit their aggregation in the brain and block the development of Alzheimer disease [4].

*Bacillus subtilis* efficiently utilizes inositol stereoisomers such as MI, DCI, and SI as carbon sources [5]. The *iolABCDEFGHIJ* operon encodes the enzymes that catabolize MI and DCI (Fig. 1). Two inositol transporters are encoded by *iolF* and *iolT* for MI and SI uptake [6, 7]. MI dehydrogenase, encoded by *iolG*, converts MI to *scyllo*-inosose (SIS) and reduces NAD^+^ in the first reaction of the catabolic pathway [8]. IolG reacts on both MI and DCI but not on SI [9]. The *iol* operon and *iolT* are regulated by the IolR transcriptional repressor, which is antagonized by the product of IolC kinase, 2-deoxy-5-keto-gluconic acid-6-phosphate [6, 10, 11]. On the other hand, the inositol dehydrogenases IolX and IolW are specific for SI and require NAD^+^ and NADP^+^, respectively [12]. Each enzyme converts SI to SIS, which is the same product generated from MI by IolG. Recently, IolU was found as the third SI dehydrogenase, which only can reduce SIS into SI in an NADPH-dependent manner [13]. Transcription of *iolX* is induced by the addition of SI to the growth medium as the sole carbon source [12]. Transcription of *iolW* is constitutive but it does not contribute to growth on SI, suggesting that IolX is essential for the catabolism of SI and that IolW is required for other reactions such as the generation of SI from SIS [5, 7].

The mechanism underlying the regulation of *iolX* to degrade SI is unknown. Within the *B. subtilis* genome, *yisR* and *iolQ* (formerly known by *degA*) reside upstream of *iolX* and are predicted to encode transcriptional regulators that belong to the AraC/XylS and LacI families, respectively (Fig. 1). Members of the AraC/XylS family include a positive regulator such as AdaA that induce the *alkA* and *ada* operons in *B. subtilis* [14]. In contrast, most members of the LacI family are negative regulators, such as CcpB [15], KdgR [16], ExuR [17], and LacR [18] in *B. subtilis*. A transcriptome analysis revealed that *yisR* and *iolQ* were transcribed from a single operon [19]. The function of YisR is unknown and its regulatory function has never been studied. On the other hand, IolQ (DegA) was named after the discovery that the recombinant form produced in *Escherichia coli* accelerated the degradation of glutamine phosphoribosyl pyrophosphate amidotransferase, implying that it might be a protease [20]. However, its sequence similarities to regulatory proteins CytR, LacI, GalR, and PurR of *E. coli* and CcpA of *B. subtilis* suggest that it could have stimulated the production of a protease [20]. In the present study, we therefore investigated the possible involvement of YisR and IolQ in the regulation of *iolX*. We show that *iolQ* encodes a transcriptional repressor that binds to the promoter region of *iolX*.

## Methods

### Bacterial strains, plasmid and growth conditions

The bacterial strains and plasmids used in this study are listed in Table 1. *B. subtilis* strain 168 is our standard strain for the study of inositol catabolism. The mutant strain BFS3018 was constructed from strain 168 and acquired from the National Bio Resource Project, National Institute of Genetics, Japan. BFS3018 has a pMUTIN4 (*lacZ lacI amp erm*) [21] integration to disrupt *iolX* which allows us to monitor *iolX* expression in an *iolX* mutated context by β-galactosidase activity [12]. The other *B. subtilis* mutant strains were constructed as described below. *E. coli* strains DH5α (Sambrook & Russell, 2001) and BL21 (DE3) (Merck Millipore) served as hosts for plasmid construction and expression of C-terminal His_6_-tagged proteins, respectively*.*

**Table 1:** Bacterial strains and plasmids

Strain or plasmid	Description	Source or reference
*E. coli*		
DH5α	*supE44 ΔlacU169* (Φ80 lacZΔM15) *hsdR17 recA1 gyrA96 thi-1 relA*	[24]
BL21	*F* ^*−*^ *ompT hsdS*_*Β*_ (r_Β_^−^m_Β_^−^) *dcm gal* (DE3) *tonA*	Merck Millipore
*B. subtilis*		
168	*trpC2*	Laboratory stock
BFS3018	*trpC2 iolX*::pMUTIN4	[12]
CM101	*trpC2* Δ*yisR*	This study
CM102	*trpC2* Δ*iolQ*	This study
Plasmid		
pMD20	TA-cloning vector, *amp*	Takara Bio
pET-30a	pET system expression vector, *kan*	Merck Millipore
pET-iolQ	pET-30 derivative to express *iolQ-His*_*6*_	This study
pET-yisR	pET-30 derivative to express *YisR-His*_*6*_	This study

*E. coli* strains were maintained in lysogeny broth (LB) medium and *B. subtilis* strains were maintained using a tryptose blood agar base (Becton Dickinson) or S6 liquid medium [22] containing 0.5% casamino acid (Becton Dickinson) and 0.005% L-tryptophan. Plasmids pMD20 (Takara Bio) and pET30a (Merck Millipore) served as vectors for TA-cloning and His_6_-tag construction, respectively. Antibiotics used as required were as follows: erythromycin (0.5 μg ml^−1^), ampicillin (50 μg ml^−1^), and kanamycin (50 μg ml^−1^). Media were supplemented with 1 mM isopropyl β-D-1-thiogalactopyranoside (IPTG) or 5-bromo-4-chloro-3-indolyl-β-D-galactoside (X-gal) as required. All bacteria were cultured at 37 °C with rotary shaking at 150 rpm.

### Construction of *B. subtilis* mutants

**Fig. 2:**
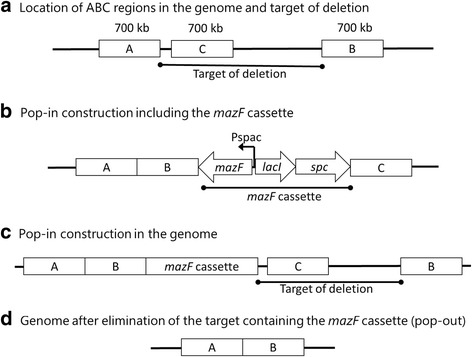
Schematic strategy of the marker-free deletion. **a** Positional relationship among the target deletion and regions A, B, and C contained in the PCR fragments used for construction of the pop-in construct. **b** Recombinant PCR pop-in construct ligating the fragments A, B, C, and the *mazF* cassette. **c** Integrant of the *mazF* cassette at the target region via a double crossover at regions A and C. An intrachromosomal crossover event between the directly repeated sequences corresponding to the region B resulted in elimination of the *mazF* cassette together with the target deletion. **d** Final structure of the marker-free deletion

CM101 (Δ*yisR*) and CM102 (Δ*iolQ*) were constructed using the marker-free approach of Morimoto et al. [23]. The pop-in construction was made by ligation of three different polymerase chain reaction (PCR) fragments amplified from the 168 genome (Fig. 2a) and another one comprising the *mazF* cassette [23]. The fragments were i) the first PCR fragment for region A located upstream of the deletion target, ii) the second for region B located downstream of the target, iii) the third for region C located inside the target, iv) and the *mazF* cassette constituted of *mazF* for suicidal toxin under the control of IPTG-inducible promoter (P*spac*), *lacI* for Lac repressor controlling P*spac*, and the spectinomycin resistance gene (*spc*). For the construction of CM101, the PCR fragments of regions A, B, C, and the *mazF* cassette were amplified using the primer pairs DyisRAF/DyisRAR, DyisRBF/DyisRBR, DyisRCF/DyisRCR, and MazFfw/MazFbw, respectively (Table 2). For CM102, the PCR fragments of regions A, B, C, and the *mazF* cassette were amplified using the primer pairs DdegAAF/DiolQAR, DiolQBF/DiolQBR, DiolQCF/DiolQCR, and MazFfw/MazFbw, respectively (Table 2). The pop-in construction containing the regions A, B, the *mazF* cassette, and region C in that order (Fig. 2b) was used to transform the parental strain 168 of *B. subtilis* for spectinomycin resistance via a double crossover in the homologous regions A and C, introducing the *mazF* cassette into the targeted region (Fig. 2c). The spectinomycin-resistant transformants were then screened on IPTG-containing plates for the detection of spectinomycin sensitive mutants. In such mutants, an intrachromosomal crossover event between the two direct repeat stretches corresponding to region B occurred to eliminate the *mazF* cassette and resulted in the marker-free deletion of the stretch between regions A and B (Fig. 2d). Correct construction of strains CM101 and CM102 was confirmed by sequencing (data not shown).

**Table 2:** Oligonucleotide primers

Primer	Sequence (5′ → 3′)*
[FAM]iolX(+50)-R†	TAACCGAGCCTTCCTAATCC
[FAM]iolX(−250)-F†	GAGCTTGTAGTCAGACATTCT
DiolQAF	TGTCAAACAGGGAACGTTAT
DiolQAR	CGCTCATTAGCGGGCCATCCCTCGTCTGGTTATTG
DiolQBF	GCCCGCTAATGAGCG
DiolQBR	CTGATTGGGTAGGATCCCCGCATGGATGGAACAGTCGATA
DiolQCF	GCTTGAGTCAATTCCGCTGTCGATGAGCTCGGTTTTCAAATG
DiolQCR	CCCATCTCTTTTATCGGCTG
iolQBamHI-R	CGCGGATCCCGTAATCGGTGCTGCAAATC
iolQEcoRI-F	GGAATTCTAACCAGACGAGGGATGAAC
iolQNdeI-F	GGGAATTCCATATGATGAAAACAACAATTTACGATGT
iolQXhoI-R	CCGCTCGAGTCATGTGTTGAGCGGTGATG
DyisRAF	TTGACAATCACAATCATCGC
DyisRAR	GTTATTGAACTTTCCGGCTGTTTTTAAGTCGGATTTTTACAAGAAG
DyisRBF	CAGCCGGAAAGTTCAATAAC
DyisRBR	CTGATTGGGTAGGATCCCCGGCATTTCTGTCGAGCAATTT
DyisRCF	GCTTGAGTCAATTCCGCTGTCGTGTCAAACAGGGAACGTTAT
DyisRCR	TCCGGTATTCAATTGGTGAA
GMSA-Nega-F	TTTTCACGGGCCGCTGCT
GMSA-Nega-R	CTCAGCATCTGGAAAATCCC
iolX (+50)-R	TAACCGAGCCTTCCTAATCC
iolX (−1)-R	GTCCCATCCTCTCCTTTATC
iolX (−200)-F	ATGAGCGGGTTTTTTCATTATG
iolX (−250)-F	GAGCTTGTAGTCAGACATTCT
MazFbw	GGGGATCCTACCCAATCAG
MazFfw	AGCGGAATTGACTCAAGC
NiolX	CGGATCGACGCTGGAGAAA
NiolXDIG	*TAATACGACTCACTATAGGG*AGCCGATAGGATGGTCACAT
PiolX400-F	TAGCCCAGCCGATAAAAGAG
PiolX400-R	TAACCGAGCCTTCCTAATCC
yisR (−1)-R	TTGAATCATCCTCCTTTTTAAGT
yisR (−200)-F	CAAGTAAGCGAAAATAATGAGAA
yisRBamHI-R	CGCGGATCCCGAGCGACAGATCCTTGATT
yisREcoRI-F	GGAATTCCTTTCTCCCGGTCTTGAACA
yisRNdeI-F	GGGAATTCCATATGATGCCTCGCATCCTGTTTAC
yisRXhoI-R	CCGCTCGAGTTATTGAACTTTCCGGCTGAC

*Restriction enzyme recognition sites and T7 RNA polymerase promoter-tag sequence are underlined and italicized, respectively

†These primers were 5′-6-[FAM]-labeled

### Enzyme assay

NAD^+^-dependent SI dehydrogenase activities in cell extracts were measured spectrophotometrically with an increase in absorbance at 340 nm with the generation of NADH as previously described [12]. β-Galactosidase activities in cell extracts were determined as previously described [25].

### RNA techniques

*B. subtilis* strains were grown at 37 °C with shaking in S6 medium containing 0.5% casamino acid, 0.005% L-tryptophan (Becton Dickinson) with or without MI or SI (10 mM each), and 10 mM glucose was added as required. Total RNAs were extracted from the cells and purified as previously described [25].

The RNA samples were subjected to a Northern blot analysis using a DIG-labeled RNA probe specific for *iolX*. The RNA probe was prepared as follows: A DNA fragment corresponding to part of the *iolX*-coding region was PCR-amplified using strain 168 DNA as a template and the primers NiolX and NiolXDIG (Table 2) to introduce a T7 RNA polymerase promoter sequence at their 3′-termini. The PCR product was used as the template for in vitro transcription using a DIG RNA labeling kit (SP6/T7) (Roche Diagnostics, Basel, Switzerland) to produce the DIG-labeled RNA probe. Cellular RNAs were separated using gel electrophoresis, transferred to a positively charged nylon membrane (Roche Diagnostics), and hybridized using the DIG-labeled probe according to the manufacturer’s instructions. Hybrids were detected using a DIG luminescence detection kit (Roche Diagnostics).

Primer extension was performed to identify the transcriptional start site of the *iolX* transcript [8]. Reverse transcription initiated from the PiolX400-R primer (Table 2) was labeled at the 5′-terminus using a Megalabel kit (Takara Bio) and [γ-^32^P]ATP (PerkinElmer). DNA from strain 168 used as the template for the dideoxy sequencing reactions, which initiated from the same end-labeled primer used for ladder preparation, was prepared by PCR using the primers PiolX400-F/PiolX400-R (Table 2).

### Plasmid construction

DNA fragments corresponding to the coding regions of *iolQ* and *yisR* were amplified from *B. subtilis* 168 genomic DNA by PCR using the respective primers iolQNdeI-F/iolQXhoI-R and yisRNdeI-F/yisRXhoI-R with generation of *Nde*I and *Xho*I sites at the 5′- and 3′-termini of each amplicon, respectively (Table 2). Each PCR product was ligated to the arms of pMD20 (Takara Bio) using a Mighty TA-cloning kit (Takara Bio) and was used to transform *E. coli* DH5α, which was then cultured on LB plates containing ampicillin, IPTG, and X-gal. White colonies were selected and plasmid DNAs were subjected to a sequence analysis using an ABI PRISM 3100 Genetic Analyzer (Thermo Fisher Scientific). The recombinant plasmids with the correct sequences were digested using *Nde*I and *Xho*I, and the restriction fragments were ligated to the arms of NdeI/XhoI-cleaved pET-30a to generate pET-iolQ or pET-yisR, which were used to transform *E. coli* BL21 (DE3) to produce C-terminal His_6_-tagged proteins IolQ-His_6_ and YisR-His_6_, respectively.

### Protein production and purification

*E. coli* BL21 (DE3) transformed with pET-iolQ or pET-yisR was inoculated into LB medium containing kanamycin and cultured at 37 °C with shaking. The recombinant proteins were induced using 1 mM IPTG when the optical density of the culture reached OD_660_ = 0.35, and the culture was further incubated for 2 h at 37 °C with shaking; the cells were harvested and disrupted by sonication. IolQ-His_6_ and YisR-His_6_ were purified from cell lysates using a TALON metal-affinity resin (Takara Bio) according to the manufacturer’s instructions.

### Gel mobility shift assay

Gel mobility shift assays were performed according to a previous study [26]. DNA fragments of the 200-bp sequences of the *iol*X and *yisR-iolQ* promoter regions were PCR-amplified using the specific primers iolX (−200)-F/iolX (−1)-R and yisR (−200)-F/yisR (−1)-R, respectively (Table 2). A negative control of a 100 bp fragment representing a segment of the *iolW* coding region was amplified using the primers GMSA-Nega-F/GMSA-Nega-R (Table 2). Each DNA fragment (0.155 pmol) was incubated in 0.02 ml of binding buffer [10 mM Tris-HCl (pH 8.0), 1 mM DTT, 10 mM KCl, 5 mM MgCl_2_, 10% glycerol, 5 μg ml^−1^ poly d(I-C), and 50 μg ml^−1^ bovine serum albumin] at 37 °C for 30 min with varying amounts of IolQ-His_6_ or YisR-His_6_. DNA protein complexes were separated using nondenaturing polyacrylamide gels in TAE buffer. The DNA fragments in the gel were stained using SYBR Green for 30 min and the bands were visualized using Chemi Doc XRS+ with Image Lab software (Bio-Rad).

### DNase I footprint assay

PCR reactions were used to amplify 5′-6-[FAM]-labeled DNA fragments containing the *iolX* promoter region (300 bp) from the DNA of strain 168 using the specific primers [FAM]iolX(−250)-F/iolX (+50)-R and iolX (−250)-F/[FAM]iolX(+50)-R for labeling the sense and antisense strands, respectively (Table 2). Each differentially 5′-6-[FAM]-labeled DNA fragment (0.45 pmol) was incubated in 0.2 ml of binding buffer with varying amounts of IolQ-His_6_ at 37 °C for 30 min. 0.75 units of DNase I (Takara Bio) was added to digest the DNA for 5 min, and the reaction was stopped by adding 0.2 ml of 0. 5 M EDTA. DNAs were extracted using a PCR purification kit (Promega). DNA sequencing of the sense and antisense strands employed the primers iolX (−250)-F and iolX (+50)-R, respectively, using the Thermo Sequenase Dye Primer Manual Cycle Sequence Kit (USB). The DNA samples were analyzed by Sigma-Aldrich using an ABI 3130xl Genetic Analyzer and ABI Gene Mapper Software Ver. 4.0 (Thermo Fisher Scientific).

## Results

### SI and MI induce the transcription of *iolX*

**Fig. 3:**
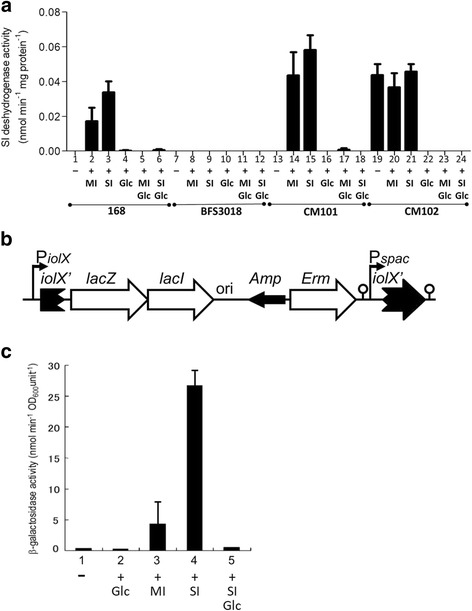
NAD^+^-dependent SI dehydrogenase activity and β-galactosidase activities of strains of *B*. *subtilis*. **a** NAD^+^-dependent SI dehydrogenase assays. Strains 168 (lanes 1–6), BFS3018 (lanes 7–12), CM101 ((Δ*yisR*, lanes 13 and 18), and CM102 (Δ*iolQ*, lanes 19–24) were inoculated into S6 medium containing 0.5% casamino acid and 0.005% tryptophan (lanes 1, 7, and 13) cultured to an OD_600_ of 1.0. As indicated, the culture media were supplemented with the carbon sources (10 mM each) MI (lanes 2, 8, 14, and 20), SI (lanes 3, 9, 15, and 21), glucose (lane 4, 10, 16, and 22), glucose plus MI (lanes 5, 11, 17, and 23), and glucose plus SI (lane 6, 12, 18, and 24). Values are means + SD obtained from three independent assays. **b** Organization of the *iolX* locus in BFS3018. **c** β-Galactosidase assays. Strain BFS3018 was inoculated into S6 medium containing 0.5% casamino acid and 0.005% tryptophan (lane 1) cultured to an OD_600_ of 0.5. As indicated, the culture media were supplemented with the carbon sources (10 mM each) glucose (lane 2), MI (lane 3), SI (lane 4), and glucose and SI (lane 5). Values are means + SD obtained from three independent assays

As shown in Fig. 3a, in the standard strain 168, NAD^+^-dependent SI dehydrogenase activity was induced in the presence of SI up to 40-fold more than its absence, while it completely disappeared in strain BSF3018 with the inactivation of *iolX* through pMUTIN4 integration (Fig. 3b). It was previously reported that BSF3018 did not grow when depending on SI as the sole carbon source [12]. In *B. subtilis*, there are at least two NADP^+^-dependent SI dehydrogenases, IolW and IolU, however neither of them functions to dehydrogenate SI to degrade it as the carbon source [12, 13]. Therefore, SI induced *iolX* to produce NAD^+^-dependent SI dehydrogenase that was responsible for the physiological utilization of SI in *B. subtilis*. Although *iolX* does not play a role in the MI catabolism [12], MI was also able to induce NAD^+^-dependent SI dehydrogenase activity up to 20-fold more than in its absence, indicating that MI also could induce *iolX* (Fig. 3a).

On the other hand, in strain BFS3018, *iolX* was inactivated but its transcription was monitored by the expression of *lacZ* for β-galactosidase activity instead (Fig. 3b). As shown in Fig. 3c, in the presence of SI and MI, β-galactosidase activity was induced up to 50- and 10-fold more than in their absence, respectively, indicating that both SI and MI are able to induce *iolX* at the transcription level without functional *iolX*. As shown in Fig. 1, SI and MI are degraded to produce the same set of intermediates [11, 12], and we can consider that none of them could be made from SI when *iolX* was inactivated, as BSF3018 did not grow when depending on SI as the sole carbon source [12]. Consequently, it is unlikely that any of the intermediates were involved in the transcriptional induction of *iolX*.

We previously reported that not only MI but also SI was mainly imported by the IolT transporter [7]. As the expression of *iolT* is controlled by IolR [6], it is thus induced when MI or SI is degraded down to the product of the IolC reaction (Fig. 1), 2-deoxy-5-keto-gluconic acid-6-phosphate, which antagonizes DNA binding of IolR [11]. Since SI can never be converted into the IolC-reaction product in BFS3018 due to the inactivation of *iolX*, the results suggest that SI uptake supported by the basal expression of *iolT* could be enough to allow induction of *iolX*. On the other hand, in BFS3018, MI is degraded involving IolG, thus allowing the induction of *iolT*. Therefore, the induction of β-galactosidase activity of BFS3018 in response to MI could be achieved due to the elevated levels of MI uptake. Nevertheless, the activity was still less than that produced in response to SI.

**Fig. 4:**
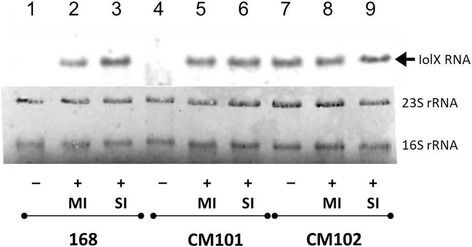
Northern blot analysis of *iolX* transcription in strains of *B*. *subtilis*. RNA samples were prepared from strains 168 (lanes 1–3), CM101 (Δ*yisR*) (lanes 4–6), and CM102 (Δ*iolQ*) (lanes 7–9), which were grown in S6 medium containing 0.5% casamino acid and 0.005% tryptophan alone (lanes 1, 4, and 7) and in the same medium supplemented with 10 mM MI (lanes 2, 5, and 8) or 10 mM SI (lanes 3, 6, and 9). The arrowhead indicates the *iolX* transcripts. The lower panel shows ribosomal RNA (16S and 23S) as the loading control

As shown in Fig. 4, the Northern blot analysis confirmed that the transcription of *iolX* in strain 168 was induced in the presence of SI or MI. The induction of NAD^+^-dependent SI dehydrogenase activity in strain 168 in the presence of SI or MI was abolished by additional glucose, suggesting that *iolX* could be under catabolite repression (Fig. 3a). In addition, the induction of β-galactosidase activity of BFS3018 in response to SI and MI was also abolished by additional glucose. These results indicatied that the induction and catabolite repression of *iolX* occurred at the transcription level (Fig. 3c).

### Expression of *iolQ* is required to regulate *iolX* transcription in response to SI

Immediately upstream of *iolX*, there is an operon that encodes two genes, *yisR* and *iolQ* [19], each of which could encode a transcriptional regulator; *yisR* and *iolQ* were predicted to encode transcriptional regulators that belong to the AraC/XylS and LacI families, respectively (Fig. 1). To determine whether YisR and IolQ regulate *iolX*, we generated the mutant strains CM101 and CM102 (Fig. 3a). In CM101 (Δ*yisR*), *yisR* was deleted to avoid the polar effect on *iolQ* downstream of it, while in CM102 (Δ*iolQ*), *iolQ* was alternatively deleted. Therefore, only *iolQ* was expressed under the control of the original *yisR*-*iolQ* promoter in CM101 whereas only *yisR* was expressed in CM102.

In CM101 (Δ*yisR*), the NAD^+^-dependent SI dehydrogenase activity of IolX was repressed in the absence of SI or MI and induced in their presence, while in CM102 (Δ*iolQ*) it became constitutive to be almost 50-fold higher than that in strain 168 in the absence of SI or MI (Fig. 3a). The activities in CM101 and CM102 in the presence of SI and MI seemed higher than those in strain 168 by unknown reasons. On the other hand, the activities in both CM101 and CM102 were repressed in the presence of glucose. These results suggest that induction of *iolX* could be regulated by IolQ but not by YisR. In addition, neither IolQ nor YisR could be involved in the catabolite repression of *iolX*.

The Northern blot analyses revealed that, in CM102 without functional *iolQ*, *iolX* was transcribed in the absence of SI and MI (Fig. 4). However, the transcription was shut off in CM101 (Δ*yisR*) when SI and MI were absent, and it was obviously induced in response to SI and MI. These results indicate that the transcriptional regulation of *iolX* in response to SI and MI depended on *iolQ* but not on *yisR*.

### IolQ binds to the *iolX* promoter region

**Fig. 5:**
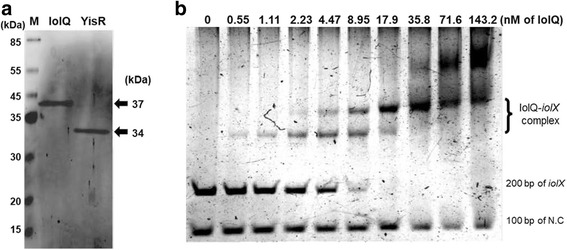
Electrophoretic gel mobility shift assay. **a** Purification of IolQ-His_6_ (IolQ) and YisR-His_6_ (YisR). The purified proteins migrated to form the respective bands in SDS-PAGE with the expected sizes (arrowheads on the right). M, size markers. **b** Results of electrophoretic gel mobility shift assay of the interaction of IolQ-His_6_ with the fragment of the *iolX* promoter. The DNA fragments corresponding to the 200 bp *iolX* promoter region (200 bp of *iolX*) and the negative control 100 bp fragment derived from the *iolW* coding region (100 bp of N. C.) were incubated with various concentrations of IolQ-His_6_ as indicated (nM of IolQ) and subjected to non-denaturing PAGE. The bands representing IolQ-His_6_-DNA complexes are indicated as the IolQ-*iolX* complex

IolQ-His_6_ and YisR-His_6_ (Fig. 5) were tested for their binding to DNA fragments containing either promoter region of the *iolX* or *yisR-iolQ* operon. Gel mobility shift assays revealed that IolQ-His_6_ formed complexes with the DNA fragment of the *iolX* promoter region (Fig. 5). The IolQ-DNA complexes formed distinct two bands, the lower and the higher molecular weight bands. As the concentrations of IolQ-His_6_ were elevated, the former appeared first at the lower concentrations, which shifted to form the latter exclusively as the concentrations increased further (Fig. 5). The results indicate that the *iolX* promoter fragment may contain at least two IolQ-binding sites with different affinities (Fig. 5); the lower molecular weight band could correspond to the IolQ-DNA complex formed by IolQ binding only to a higher affinity site while the higher molecular weight one was formed by its binding to both higher and lower affinity sites. Neither SI nor MI (at higher concentrations up to 20 mM) affected the specific DNA binding of IolQ-His_6_ in vitro (data not shown). In addition, another set of gel mobility shift experiments involving not only IolQ-His_6_ but also YisR-His_6_ was conducted. Nevertheless, neither SI, MI, nor SIS caused any effect on DNA binding of IolQ-His_6_ in the additional presence of YisR-His_6_ (data not shown).

On the other hand, IolQ did not interact with the *yisR*-*iolQ* promoter region, and we failed to detect YisR-His_6_ binding to either fragment of the *iolX* or *yisR*-*iolQ* promoter region in the presence and absence of any of MI, SI, and SIS (data not shown).

### Identification of the two IolQ-binding sites within the *iolX* promoter region

**Fig. 6:**
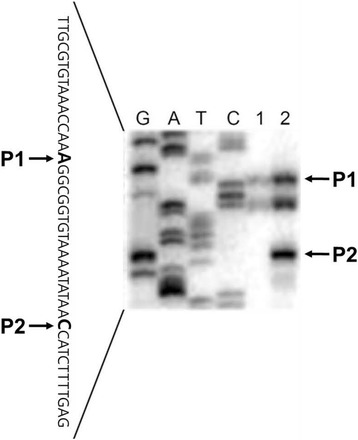
Primer extension analysis of the *iolX* transcript. Total RNA samples extracted from strain 168 grown in S6 media containing 0.5% casamino acid, 0.005% tryptophan, not supplemented (lane 1) or with 10 mM SI (lane 2) were reverse transcribed to generate cDNA. Lanes G, A, T, and C are dideoxy sequencing ladders that correspond to the reverse transcript (lower strand) generated from the same primer used for the reverse transcription. The partial nucleotide sequence of the upper strand of the promoter region is shown on the left where the identified two 5′-ends of the transcripts from the promoters P1 and P2 are indicated in bold face, whereas the reverse transcripts corresponding to the promoter P1 and P2 are indicated by arrowheads on the right side

**Fig. 7:**
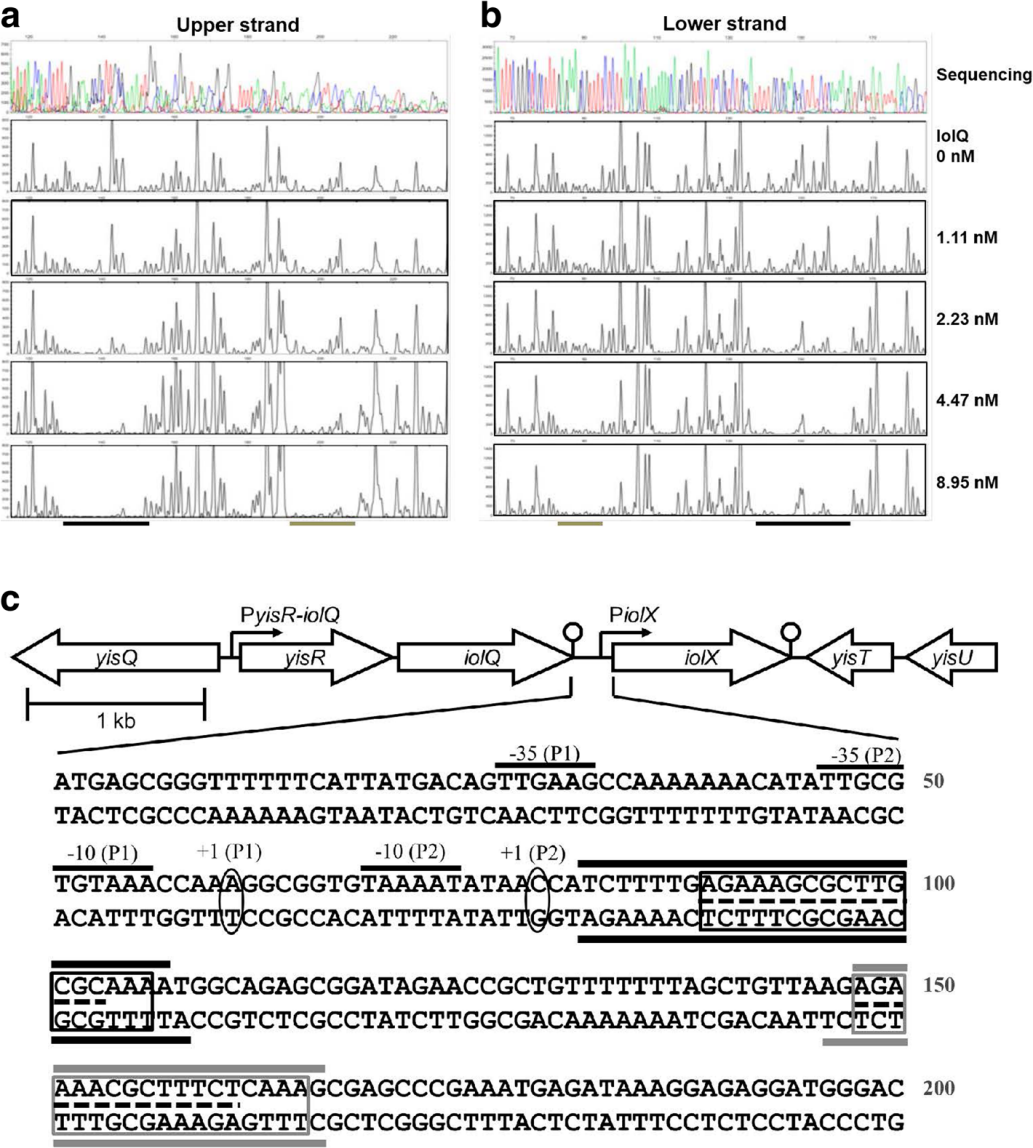
DNase I foot printing of IolQ-His_6_ on the *iolX* promoter region. DNase I foot printing of the upper **a** and lower **b** strands. Sequence data are shown on the top and below are fragment analysis data acquired using various concentrations of IolQ-His_6_ as indicated on the right. **c** Summary of DNase I foot printing data. The nucleotide sequences (upper and lower strands) of the DNA fragment that correspond to the 200 bp *iolX* promoter region used for the electrophoretic gel mobility shift assay are shown. Transcription initiation sites +1 (P1) and +1 (P2) and their corresponding −35 and −10 regions are indicated. The protected regions with higher and lower affinities are indicated by black and gray bars, respectively. The conserved sequences within the protected regions are boxed. The *cre* sites are indicated by the dashed bars between the upper and lower strand sequences within the two regions for IolQ binding

The primer extension experiment (Fig. 6) determined two transcriptional start sites downstream of the promoters P1 and P2 for the *iolX* transcript. Only a small amount of the reverse transcript corresponding to promoter P1 was detected in the absence of SI, but it was significantly induced in response to SI together with the additional transcript corresponding to P2. Their respective −35 and −10 regions were deduced to serve as the *iolX* promoters P1 and P2 (Fig. 7). Another reverse transcript was found to be as strong as the one corresponding to promoter P1 but was shorter by 6 bp. This was considered to be due to a truncated product derived from the P1 transcript, since there are no consensus −35 and −10 sequences corresponding to this 5′ end.

IolQ-binding sites within the *iolX* promoter region identified using a DNase I footprint analysis revealed that IolQ bound with different affinities to the two regions (Fig. 7). The stretches with sequences TCTTTTGAGAAAGCGCTTGCGCAAAAT (spanning +4 to +30 bp, position numbers assigned relative to the transcription start site of the promoter P2) and AGAGAAAACGCTTTCTCAAAG (spanning +68 to +88 bp) were protected from DNase I at lower and higher concentrations of IolQ, respectively (Fig. 7). Therefore, the former and the latter stretches were judged as the higher and lower affinity regions, respectively. The two protected regions contained the conserved sequence AGAAARCGCTTKCKCAAA (where R = A or G and K = G or T), which may represent a core recognition sequence required for IolQ binding. The protected stretch of the higher affinity region extended 7 bp upstream and 1 bp downstream compared with that of the lower affinity site. Previously, a plausible *cre* site for CcpA/P-Ser-HPr binding was predicted in the *iolX* promoter region [27], which was found to be overlapping the lower affinity region and was supposed to be involved in catabolite repression (Fig. 7). In addition, we could also predict another plausible *cre* site within the higher affinity region.

## Discussion

*B. subtilis* strains possess at least three types of SI dehydrogenases encoded by *iolX*, *iolW* [12], and *iolU* [13]. IolX requires NAD^+^ and both IolW and IolU need NADP^+^ as a cofactor. It is known that *iolX* plays an indispensable role in the utilization of SI as a carbon source for growth [12], and we showed here that *iolX* was induced more than 40-fold in the presence of SI (Figs. 3 and 4). The transcription of *iolW* is constitutive, and IolW can convert SI into SIS in vitro but does not contribute to growth depending on the availability of SI as the carbon source [12]. IolU is also produced constitutively and generally at low levels [19] and was not able to dehydrogenate SI but only reduce SIS into SI [13]. We hypothesized that *yisR* and *iolQ*, which are located and cotranscribed [19] immediately upstream of *iolX*, might encode the regulator(s) of *iolX* transcription (Fig. 1). YisR is a member of the AraC/XylS family, which includes mainly positive transcription regulators [13], and IolQ is a member of the LacI family of negative transcription regulators [28], which contain the typical helix-turn-helix motif, characteristic of a DNA-binding domain [29]. The present results suggested that YisR was unlikely to be involved in the regulation of *iolX* transcription (Figs. 3 and 4). Usually, the regulatory function of AraC/XylS family members requires specific cofactors; for example, *B. subtilis* Btr needs binding with its co-activator, the siderophore bacillibactin, to exert its regulatory function [30]. Therefore, we hypothesized that one of MI, SI, and SIS might be a cofactor of YisR, but none of them enhanced YisR-His_6_ binding to the *iolX* and *yisR*-*iolQ* promoter regions. On the other hand, since the DNA binding motif of AraC family proteins is near the C-terminus, the C-terminal His-tag fusion of YisR-His_6_ could affect DNA binding. Obviously, further studies are required to clarify transcriptional regulation involving YisR.

The data presented here indicate that *iolQ* encodes a repressor that binds to two sites within the *iolX* promoter region (Figs. 5 and 7). In addition, the repression is released in the presence of SI or MI (Figs. 3 and 4). *iolX* encodes NAD^+^-dependent SI dehydrogenase that is responsible for physiological SI catabolism [12]. Even when we functionally inactivated *iolX* in BF3018 by inserting pMUTIN4, the transcription of *iolX-lacZ* was prominently elevated in media containing SI (Fig. 3c). We considered the possibility that the inducing signal was a derivative of SI not requiring IolX for its synthesis. However, we failed to identify any good candidates. Although IolW is constitutively produced, it only inefficiently coverts SI into SIS with the predominating reverse reaction [12]. We previously demonstrated that MI was converted into SI through the coupling reactions involving IolG and IolW; the former dehydrogenates MI into SIS with a reduction of NAD^+^, and the latter reduces SIS into SI with oxidation of NADPH [5]. However, the conversion was detected only when the intermediate SIS was accumulated by the additional inactivation of *iolE*, which encodes SIS dehydratase acting on SIS for further degradation of this intermediate (Fig. 1) [5]. Another NADP^+^-dependent SI dehydrogenase encoded by *iolU* was recently identified [13]. Although this enzyme is not as active as IolW, it is able to convert SIS into SI but only when overexpressed. Therefore, IolU is unlikely to be involved in the possible conversion of MI into SI. All of these observations led us to speculate that mainly SI and secondarily MI could be the intracellular inducers interacting with IolQ to antagonize its DNA binding, allowing the induction of *iolX*, however they failed to antagonize DNA binding of IolQ-His_6_ in vitro. The C-terminal His-tag fusion might affect effector binding.

We showed here that IolQ bound with different affinities to the two sites within the *iolX* promoter region. The high affinity site was located from positions +4 to +30 of the promoter P2 within the sequence TCTTTTGAGAAAGCGCTTGCGCAAAAT, and the low affinity site was located from +68 to +88 within the sequence AGAGAAAACGCTTTCTCAAAG (Fig. 7). Most members of the LacI family preferentially require a palindromic sequence within their DNA binding sites [28]. A comparison between the sequences of the two IolQ binding sites identified the relatively conserved sequence AGAAARCGCTTKCK, which may suggest the potential perfect palindrome could be AGAAAGCGCTTTCT. However, this perfect palindrome is not present in either of the two binding sites that differ in two and one positions in the higher and lower affinity binding sites, respectively. Therefore, the consensus palindrome is not the only determinant of IolQ binding, although the sequences extending from the conserved stretch may contribute to high affinity binding of IolQ to its target sequence. Within the *B. subtilis* genome, there are 22 sites with a sequence similar to the conserved consensus sequence (maximum of two different positions, data not shown). At least seven of the 22 sites are located close to promoter regions, including the one of the *iolX* promoter. Thus, IolQ may regulate six additional promoters and therefore drive the transcription of at least the following genes (products): *glpT* (glycerol-3-phosphate permease), *ycsA* (putative enzyme similar to 3-isopropylmalate dehydrogenase), *acoR* (transcriptional activator of acetoin utilization genes), *yrbE* (another member of the Gfo/Idh/MocA family paralogs including *iolG*, *iolU*, *iolW*, and *iolX*) [13], *menA* (1,4-dihydroxy-2-naphthoate octaprenyltransferase), and *bglS* (endo-β-1,3–1,4 glucanase). Our future course will focus on determining the mechanisms of transcriptional regulation of these genes and their involvement in SI metabolism.

Expression of *iolX* for NAD^+^-dependent SI dehydrogenase activity in strain 168 as well as the β-galactosidase activity in strain BFS3018 was almost completely repressed in response to glucose even in the presence of SI and MI, indicating that *iolX* is under catabolite repression (Fig. 3a and c). The plausible *cre* site predicted as overlapping the lower affinity region for IolQ binding (Fig. 7) might be involved in catabolite repression. We noticed that part of the conserved sequence AGAAARCGCTTKCK for IolQ binding was quite similar to the one WGNAANCGNTTNCW for CcpA/P-Ser-HPr biding [31]. In addition, the sequence AGAAAGCGCTTGCGC within the higher affinity site for IolQ binding was also similar to the *cre* site consensus (Fig. 7). Both or either of the two IolQ-binding sites might also function as the binding site of CcpA/P-Ser-HPr in the presence of glucose. Since *iolX* functions for the catabolism of SI as a minor alternative carbon source, it makes sense that this gene is regulated by global catabolite repression involving CcpA/P-Ser-HPr [31].

## Conclusion

In *B. subtilis*, both SI and MI induce *iolX* expression for NAD^+^-dependent SI dehydrogenase activity. The *iolX* expression became constitutive in an *iolQ* background, and IolQ binds to two sites upstream of *iolX* where two transcription start sites were located. Genetic evidences allowed us to speculate that SI and MI might possibly be the intracellular inducers; however they failed to antagonize DNA binding of IolQ in in vitro experiments.

## Abbreviations

DCI: D-*chiro*-inositol
IPTG: isopropyl β-D-1-thiogalactopyranoside
LB: lysogeny broth
MI: *myo*-inositol
*o*-NP: *o*-nitrophenol
PCR: polymerase chain reaction
P*spac*: *spac* promoter
SI: *scyllo*-inositol
SIS: *scyllo*-inosose
X-gal: 5-bromo-4-chloro-3-indolyl-β-D-galactoside.
